# Elucidating Grinding Mechanism by Theoretical and Experimental Investigations

**DOI:** 10.3390/ma11020274

**Published:** 2018-02-09

**Authors:** AMM Sharif Ullah, Alessandra Caggiano, Akihiko Kubo, M. A. K. Chowdhury

**Affiliations:** 1 Faculty of Engineering, Kitami Institute of Technology, 165 Koen-cho, Kitami 090-8507, Japan; kuboak@mail.kitami-it.ac.jp; 2 Department of Industrial Engineering, University of Naples Federico II, P.le Tecchio 80, 80125 Naples, Italy; alessandra.caggiano@unina.it; 3 Industrial Engineering Department, College of Engineering, King Saud University, P.O. Box 800, Riyadh 11421, Saudi Arabia; mchowdhury@ksu.edu.sa

**Keywords:** multi-pass grinding, brittle materials, grinding wheel, surface topography, complexity

## Abstract

Grinding is one of the essential manufacturing processes for producing brittle or hard materials-based precision parts (e.g., optical lenses). In grinding, a grinding wheel removes the desired amount of material by passing the same area on the workpiece surface multiple times. How the topography of a workpiece surface evolves with these passes is thus an important research issue, which has not yet been addressed elaborately. The present paper tackles this issue from both the theoretical and the experimental points of view. In particular, this paper presents the results of experimental and theoretical investigations on the multi-pass surface grinding operations where the workpiece surface is made of glass and the grinding wheel consists of cBN abrasive grains. Both investigations confirm that a great deal of stochasticity is involved in the grinding mechanism, and the complexity of the workpiece surface gradually increases along with the number of passes.

## 1. Introduction

**Figure 1:**
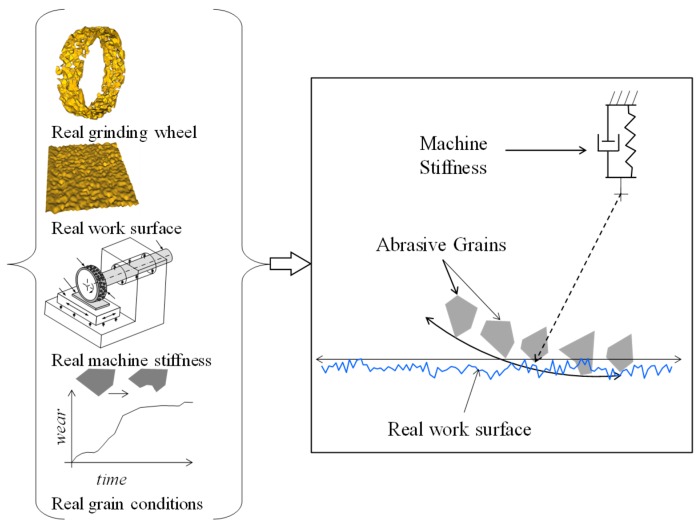
Reality in grinding and microscopic interaction between grinding wheel and workpiece surface.

A manufacturing process called grinding (which accounts for about 20–30% of the total expenditure on machining operations in industrialized nations) is often used to machine the technical ceramics (e.g., optical glass and carbides) and metallic materials with the high surface finish, surface integrity, and dimensional/form tolerance. In grinding, the abrasive grains (e.g., cBN, diamond, silicon carbide, alumina, and so on), which are by nature very hard and brittle, attached on a grinding wheel remove the required amount of materials from a workpiece surface very slowly [1,2,3]. As far as the wear behavior of the abrasive grains is concerned, different types of abrasive grains exhibit very similar wear patterns (i.e., micro-nano level cracks are generated due to the cutting action resulting in the sudden fracture and loss of the cutting edge) [2] As a result, loss and truncation (wear) of abrasive grains are the common phenomena associated with grinding. At the same time, uneven size and distribution of abrasive grains are the integrated parts of grinding. These facts result in a very complex microscopic interaction between the abrasive grains (attached on the outermost circumference of a grinding wheel) and workpiece surface, as schematically illustrated Figure 1. In addition, the following aspects contribute to this complex interaction: roughness of the workpiece surface, vibrations generated due to machine and grinding wheel stiffness, form errors associated with the workpiece and grinding wheel (not shown in Figure 1), thermal, elastic, and plastic deformations of grinding wheel and workpiece (not shown in Figure 1). Many authors have studied the abovementioned complex interaction under the umbrella called grinding mechanism. A relatively comprehensive literature review on the grinding mechanism is presented in the following section.

However, in most real-life applications, a grinding wheel passes the same area on the workpiece surface multiple times (hereinafter referred to as multipass grinding) to remove the required amount of materials because a single pass may not be sufficient. As reported in the literature review section (Section 2), the studies done so far on the grinding mechanism do not address the issue of multipass grinding. This means that how the topography of a workpiece surface evolves with the increase in the number of passes is somewhat unknown. Thus, elucidating grinding mechanism from the perspective of multipass grinding deserves investigations. Since all real-life factors affecting the topography of a ground surface are not possible to accommodate in a theoretical model, experimental investigations should be carried out (besides the theoretical modeling of grinding mechanism) to have a relatively comprehensive picture. From this contemplation, this paper is written. Accordingly, this paper addresses the mechanism of multipass grinding. In particular, the results of theoretical modeling and experimental investigations on multipass surface grinding operations are presented. For obtaining the experiment results, a workpiece made of glass is surface ground using a grinding wheel that consists of cBN abrasive grains. It is worth mentioning that the objective of conducting experiments is not to validate the theoretical model of grinding mechanism but to see the overall trend of the (multipass) grinding mechanism from both perspectives.

The remainder of this paper is organized as follows: Section 2 provides a literature review on the grinding mechanism elucidating the governing parameters that control the workpiece surface topography. Section 3 describes a preliminary experimental investigation on the multipass grinding so see how the topography of a ground surface evolves with the number of passes from the qualitative viewpoint. Section 4 describes a theoretical model of grinding mechanism as well as a computing tool that implements the model. The pass-by-pass evolution of the ground surface topography is also reported in this section from the theoretical perspective. Section 5 describes the experimental investigations showing how the surface topography evolves due to multipass grinding from the real-life perspective. Section 6 provides the concluding remarks of this paper.

## 2. Literature Review on Grinding Mechanism

Similar to the mechanisms of other material removal processes, the grinding mechanism has been studied by numerous authors. The studies done in the early 1950s–1960s on grinding mechanism focused on the Euclidian geometry-based interaction between abrasive grains and workpiece surface wherein the main concern was to elaborate on the uncut chip thickness and nominal surface topography of the ground surface. Later, the trend has changed; along with the Euclidian geometry-based analysis, stochastic processes have started to play their roles. This trend even continues up to now. Some of the selected studies are briefly described, as follows:

Matsui and Syoji [4,5,6] and Matsui [7] developed grinding mechanism models accommodating the uneven 3D distributions of cutting edges of the abrasive grains in a stochastic manner for determining the surface roughness, maximum grain depth of cut, and uncut chip length, and alike under various grinding conditions. The authors also identified the influences of the positions and tip angles of cutting edges on the grinding performances. Matsui and Tamaki [8] developed a grinding mechanism simulation system incorporating the elastic and plastic deformations of a ground surface. The simulation results and the experimental results of grinding force and ground surface roughness show a close similarity. Shimada et al. [9] modified the models of Matsui and Syoji [4,5,6] and Matsui [7] and proposed a theoretical model of grinding mechanism for calculating the surface roughness for the slant feed grinding operations. The calculated results were validated by experimental ones using a customized vibration assisted micro-grinding device. Hecker and Liang [10] predicted the surface roughness of a workpiece surface due to grinding based on the probabilistic un-deformed chip thickness model, which includes such parameters as microstructure of grinding wheel, kinematic conditions, and material properties of workpiece surface. The simulated surface roughness showed a close agreement with the experimental data obtained by performing cylindrical grinding. The model also described the effects of such grinding parameters as depth of cut, speed ratio, equivalent diameter, and wheel microstructure on the surface roughness. Agarwal and Rao [11] developed an analytical surface roughness model based on stochastic nature of the grinding, which was governed by the random geometry and random distribution of the abrasive grains. The model was validated by experimental results of surface roughness of a workpiece surface made of silicon carbide. Nguyen and Butler [12] developed a kinematic simulation model of grinding using an algorithm. To run the model, one needs to identify the active number of abrasive grains and their attack angles estimated from the wheel topography. The estimated attack angle determines whether the grain will cut, plow, or rub the workpiece surface. The experimental results verified the effectiveness of the method. The results were obtained by using a grinding wheel made of alumina and a workpiece made of mild and tool steels. Chakrabarti and Paul [13] developed a numerical simulation technique to generate the grinding wheel topography using square pyramidal grains. The ground workpiece surface was also generated simulating the trajectory of all the abrasive grains and removing the interfering material. The effects of different grinding parameters on the average surface roughness of the generated workpiece have been studied. Finally, the variation of surface roughness with the maximum uncut chip thickness was studied. Nguyen and Butler [14] showed that the characteristics of the grinding wheel topography using the three-dimensional surface characterization parameters (e.g., root-mean-square roughness), and showed how the characteristics varies with the density and sharpness of abrasive grains and coarseness of the grinding wheel. Oliveria et al. [15] described the influences of vibration in grinding for a specific case of low stiffness parts of DTG materials. The results show that induced random vibration, such as white noise, affects the wheel action reducing the grinding power and increasing wheel wear. Chatter tests showed the phenomenon where intense chatter quickly progresses around the wheel surface from a starting point. Li and Rong [16] presented a kinematic simulation model of grinding to find out the number of contacting abrasive grains, contact cross-sectional area for each grain and resultant workpiece surface area under given grinding conditions. The model can calculate both cutting and plowing forces. Heinzel and Rickens [17] focused on the evaluation of topographical parameters affecting the material removal process in grinding of optical glass by engineered diamond wheels. Two topographical parameters, the specific total grain plateau and the average grain cutting edge width, determined by 3D-profilometry of replicated abrasive layers after each dressing step, did the characterization of the abrasive layer topography. These parameters directly characterized the topographical condition of the active abrasive layer and therefore the grinding capability of the diamond grinding wheels. Durgumahanti et al. [18] developed a model for predicting the total grinding force by taking into account the combined effect of the coefficient of friction and plowing force of abrasive grains. In this model, the coefficient of friction varies with the process parameters. A single-grain scratch test was adopted for developing the plowing force model. The predicted normal and tangential grinding forces showed a good agreement with the experimental results. Stepien [19] used the grinding wheels having (single and double) helical grooves to generate the regular surface textures and found that the surface texture has two components, namely, deterministic component resulting from the nominal wheel active surface and stochastic component resulting from the random geometry and arrangement of abrasive grains. Furthermore, the author revealed that the influence of the stochastic component limits the surface generation process. Aurich and Kirsch [20] developed a kinematic simulation model of grinding to compute and evaluate the chip parameters of each grain participating in the material removal process. This model is useful for investigating the influences of process parameters and the grinding wheel topography on the material removal process. Jiang et al. [21] developed a numerical model of grinding mechanism that describes the micro-interaction situations in the contact zone between abrasive grains and workpiece surface where the contact zone was divided into four categories, namely, no-contact, sliding, plowing, and cutting grains. In this model, interaction between grain and workpiece surface was determined by two parameters, namely the grain penetration depth and the grain diameter. Different distributions were used to achieve the abovementioned interactions. The surface roughness predicted by the model showed good agreement with the experimental data. Darafon et al. [22] developed a stochastic model of grinding mechanism where the uncut chip thickness and the contact length of the abrasive grains were calculated to predict the instantaneous material removal rate and surface roughness of the workpiece surface. The authors reconfirmed that the percentage of active abrasive grains and the geometry of grinding chip (length and thickness) found in the reality do not match with those suggested by the Euclidian geometry-based analytical models developed in 1950s–1960s. Sousa et al. [23] focused on the micro- and macro-kinematics of the grinding operation for machining glass. Two different arrangements of abrasive grains were tested under three different kinematic curves, eliciting the effect of the abrasive grains on the surface topography. To study the influences of the grinding on the workpiece surface topography, a simulation model for the surface topography was proposed by Cao et al. [24], in which both the grinding wheel surface topography and the relative vibration between grinding wheel and workpiece were considered. The simulation results showed the influence of grinding wheel vibration amplitude, number of abrasive grains, as well as the process variables on the surface waviness and roughness. Chen et al. [25] investigated the effects of vibration of the grinding system on the relationship between surface roughness and subsurface damage based on grinding kinematics analysis and indentation fracture mechanics, with the aim to improve the prediction accuracy of surface roughness and subsurface damage, especially in micromachining of brittle materials. Osa et al. [26] presented a numerical model to simulate the contact between grinding wheel and workpiece in surface grinding, reproducing the granular structure of the grinding wheel using the discrete element method. The surface topography is applied on the model surface taking into account the dressing mechanisms and movements of a single-point dresser. The individual contacts between abrasive grits and workpiece are studied regarding the uncut chip thickness, assuming visco-plastic material behavior. The results remark the importance of surface topography and dressing conditions on the contact area, as well as wheel deflection. In Wang et al. [27], a simulation method was proposed to visualize the grinding surface topography and predict grinding surface roughness. It was shown that at small cutting depth the surface microstructure could be improved up to a certain value of the ratio between the cutting velocity and feed rate. Incorporating vibration analysis in the modeling further proved the theory of topography analysis. McDonald et al. [28] developed a grinding mechanism model where the model parameters are refined using the grinding wheel topography data. It can predict the uncut chip thickness with high precision and the roughness of the workpiece surface that is the result of the interactions of many unevenly distributed abrasive grains. Ding et al. [29] conducted grinding wheel wear experiments to investigate the evolution and influence of the grain protrusion height non-uniformity. Grinding wheel topography reconstruction was performed using the un-deformed chip thickness non-uniformity investigation, ground surface roughness model. Ullah et al. [30] developed a stochastic simulation model of grinding mechanism where the trajectories of the stochastically distributed abrasive grains with uneven heights and the roughness of the workpiece surface are integrated to see how the workpiece surface topography evolves due to multiple passes.

Apart from the grinding operation along the effect of dressing is also incorporated in the grinding mechanism. For example, Jiang et al. [31] developed a 2D and 3D ground surface topography models based on the microscopic interaction between abrasive grains and workpiece surface. In addition to grinding parameters, wheel dressing (dressing depth, dressing lead, geometry of diamond dressing tool) and wear effects of both wheel and diamond dressing tool were considered in this study. The developed model was verified by the results of surface grinding experiments where the workpiece was made of hard steel and the grinding wheel was made of alumina (vitrified bonded). Chowdhury et al. [32] developed a kinematic model of dressing mechanism that alters the surface topography of a grinding wheel, and, thereby the workpiece surface. The effect of multiple passes was also elucidated for different dressing conditions. Kubo et al. [33] showed the effect of the rotary diamond dresser while dressing a grinding wheel under different dressing conditions. In this study, the 3D effect of dressing grit trajectories on the grinding wheel topography was elucidated using simulation. This study also reports the effect of multiple passes on the grinding wheel topography.

From the above descriptions, it is clear that numerous authors have been trying to develop the methodologies and systems to understand, as well as to predict, the consequences of the microscopic interactions that takes place between the abrasive grains of grinding wheel and workpiece surface, in presence of the following conditions: loss/truncation/wear of abrasive grains, uneven size, height, and distribution of abrasive grains, roughness of the already-ground workpiece surface, thermal/elastic/plastic deformations, and machine/grinding wheel stiffness. However, the pass-by-pass evolution of the workpiece surface topography has not yet been reported from the viewpoints of both experimental and theoretical investigations.

## 3. Preliminary Experimentation

**Figure 2:**
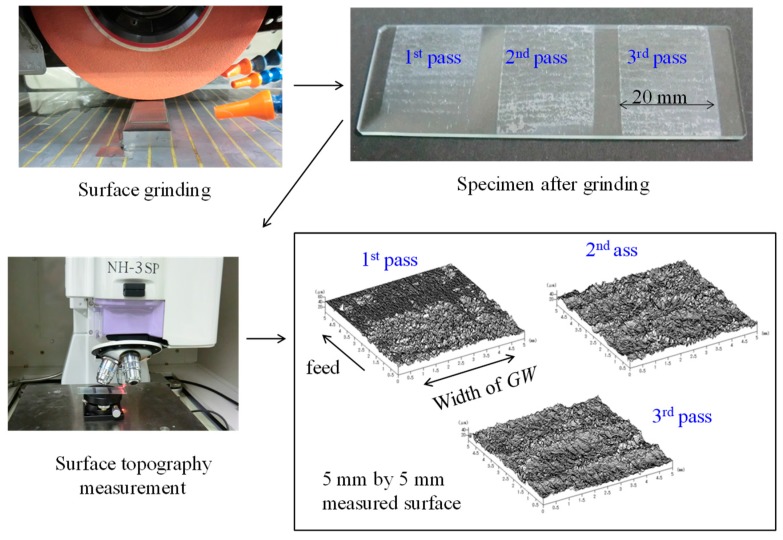
Results of a preliminary experiment.

To understand the reality of the multipass grinding operations from the quantitative perspective, a set of preliminary experiments have been conducted. The section describes the salient points of these experiments using the results of one of the experimentations, as shown in Figure 2.

As seen from Figure 2, a rectangular specimen made of glass (26 mm × 75 mm × 1.5 mm) was surface ground using a grinding wheel (GW) denoted as WA120N7V58R (Noritake Co., Ltd., Nagoya, Japan). The GW diameter and width were 200 mm and 20 mm, respectively. The GW’s surface speed was 31.4 m/s and the table feed was 0.1 m/s. Three different areas on the specimen were selected for performing multipass grinding where the depth of cut was 2 μm for all passes. The top-right segment of Figure 2 shows these three areas on the specimen after the first, second, and third pass, respectively. The segment marked 1st pass (see Figure 2) was ground only once. The segment marked 2nd pass was ground twice. The segment marked 3rd pass was ground three times. The surface heights were measured using a non-contact surface metrology instrument, as shown in Figure 2. The visual inspection of the ground surface and the surface topography shown in Figure 2 reveals that the topography of the ground surfaces is very complex and evolves with the pass; each additional pass helps remove more materials from the workpiece surface. As a result, the surface becomes deeper and deeper with the increase in the number of passes. The microscopic interaction between the abrasive grains of the grinding wheel and the workpiece surface results in an uneven material removal from the workpiece surface. This interaction can be studied from both theoretical and experimental viewpoints, as it is described in the next two consecutive sections.

## 4. Theoretical Model of Grinding Mechanism

**Figure 3:**
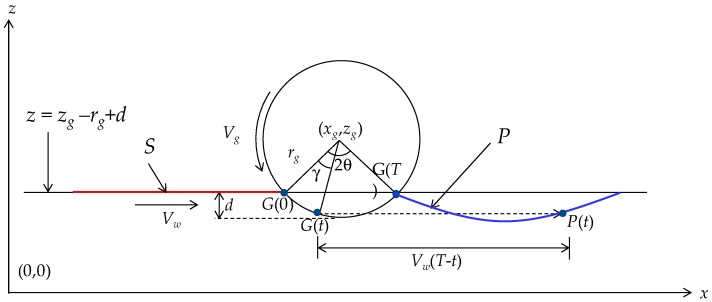
Grinding by a single grain.

As described in the literature review section (Section 2), numerous authors have worked on the issue of developing a reliable model of grinding mechanism that accurately mimics the microscopic interaction between the abrasive grains and workpiece surface, creating a complex workpiece surface (see Figure 3). Referring to the relevant works (as described in Section 2), Ullah et al. [30] proposed a set of nine functional requirements needed for developing a realistic model that mimics the microscopic interaction between the abrasive grains and workpiece surface. The main goal of these functional requirements is to create a resultant trajectory from the trajectories of some unevenly distributed abrasive grains with uneven heights. The trajectories for a pass interact with the trajectories of the previous pass. This way, the topography of the workpiece surface evolves. The following subsections define a procedure to determine the trajectories of some stochastically distributed abrasive grains having stochastic heights. A computer-based grinding mechanism simulation tool is also presented in this section that integrates the outcomes of analyses described in the remainder of this section.

### 4.1. Effect of a Single Grain

Figure 3 schematically illustrates the interaction between a point (an abrasive grain) attached on the outermost circumference of a grinding wheel and the workpiece surface. The mathematical description of this interaction is as follows. Let ($\underset{-}{x_{g}}$,$\underset{-}{z_{g}}$) be the coordinates of the center of the grinding wheel having a (nominal) radius $\underset{-}{r_{g}}$ and diameter *d_g_* (i.e., *d_g_* = 2*r_g_*) on the $\underset{-}{xz}$-plane. Let *V_g_* be the surface velocity of the grinding wheel. Let $\omega_{g}$ be the rotational speed of the grinding wheel, i.e., $\omega_{g}r_{g}=V_{g}$. Let *V_w_* be the workpiece surface velocity that acts in the *x*-direction. The case shown in Figure 3 corresponds to a cut called down-cut because *V_w_* and *V_g_* are in the same direction. The opposite case is called up-cut. Let *d* be the depth of cut of the grinding operation. Let *S* be the segment of workpiece surface that will be ground by the grain given by a point called *G* on the outermost circumference of the grinding wheel. This means that *G* gradually grinds *S* and lefts a trajectory *P* on the workpiece surface. This means that *S* becomes *P* due to the grinding action of *G*. As a result, the area confined by the trajectory *P* and a line *z* = *z_g_* − *r_g_* + *d* is the area from where the materials will be removed due to the action of the abrasive grain *G*. To be more specific, let *G*(*t*) be the position of the abrasive grain at time *t*. Let *T* be the contact period of the abrasive grain, i.e., *t* ∈ [0, T].

Thus, *G*(0) and *G*(*T*) are the initial and final positions of the abrasive grain, respectively. Let 2*θ* be the attack angle of the abrasive grain. If the attack angle is very small, which is the case for grinding due to a very small depth of cut compared to the diameter of the grinding wheel (*r_g_* > *d*), it can be expressed as follows:

(1) $$\theta =\left(\sqrt{\frac{2d}{r_{g}}}\right)$$

Thus, the contact period *T* is given as follows:

(2) $$T=\frac{2\theta r_{g}}{V_{g}}=2\frac{\sqrt{2dr_{g}}}{V_{g}}$$

Now, a point on *S* at time *t* makes contact with the grain and takes its position at *G*(*t*) = (*G_x_*(*t*),*G_z_*(*t*)) due to the cutting action of the grain. Afterwards, it (i.e., the point on *S* that contacts *G*) travels in the *x*-direction for the rest of the time (*T* − *t*) at the speed of workpiece, *V_w_*. This creates the trajectory *P*(*t*) = (*P_x_*(*t*),*P_z_*(*t*)), as follows:

(3) $$P_{x}\left(t\right)=G_{x}\left(t\right)+V_{w}\left(T-t\right)=x_{c}-r_{g}\sin\left(\theta -\omega_{g}t\right)+V_{w}\left(T-t\right)$$

(4) $$P_{z}\left(t\right)=G_{z}\left(t\right)=z_{c}-r_{g}\cos\left(\theta -\omega_{g}t\right)$$

It is worth mentioning that in Equations (3) and (4) *t* no longer plays the role of time; it is just a parameter that helps locate a point on the grinding trajectory *P*. This means that *t* = 0 represents the end point of the trajectory *P*, i.e., *P*(0), and *t* = *T* represents the starting point of the trajectory of *P*, i.e., *P*(*T*).

### 4.2. Effect of Two Consecutive Grains

**Figure 4:**
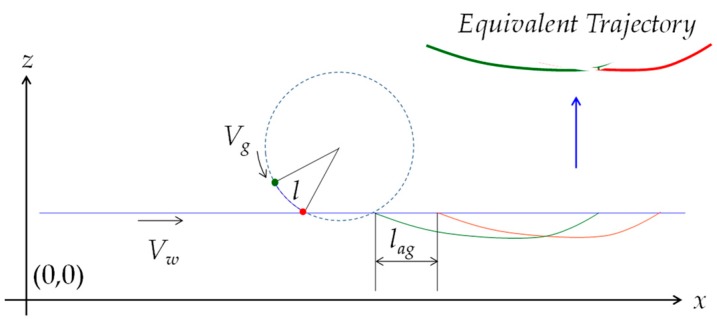
The concept of equivalent trajectory.

Consider that there are two grains on the outermost circumferential surface of the workpiece, as shown in Figure 4. Let the circumferential distance between the grains be *l*. As schematically illustrated in Figure 4, there is a time, denoted as *δ*, between the trajectories of the first and the last grains, as follows:

(5) $$\delta =\frac{l}{V_{g}}$$

The distance between the trajectories denoted as *l_ag_* is given, as follows:

(6) $$l_{ag}=V_{w}\delta =l\frac{V_{w}}{V_{g}}=lV_{r}$$

In Equation (6), *V_r_* denotes the velocity ratio (*V_w_*/*V_g_*). Therefore, the trajectory of the first grain, denoted as *P*_1_(*t*), is given by:

(7) $$P_{x1}\left(t\right)=x_{c}-r_{g}\sin\left(\theta -\omega_{g}t\right)+V_{w}\left(T-t\right)+lV_{r}$$

(8) $$P_{z1}\left(t\right)=z_{c}-r_{g}\cos\left(\theta -\omega_{g}t\right)$$

As such, the trajectory of the last or second grain denoted as *P*_2_(*t*) is given, as follows:

(9) $$P_{x2}\left(t\right)=x_{c}-r_{g}\sin\left(\theta -\omega_{g}t\right)+V_{w}\left(T-t\right)$$

(10) $$P_{z2}\left(t\right)=z_{c}-r_{g}\cos\left(\theta -\omega_{g}t\right)$$

For some values of *t*, denoted as *t*_1_, *t*_2_ ∊ [0, T], the *x*-coordinates of the trajectories can be the same. In this case, the minimum value of the *z*-coordinates of the trajectories is the effective *z*-coordinate of the trajectories. This refers to a condition called equivalent trajectory condition, as follows:

(11) $$P_{x1}\left(t_{1}\right)=P_{x2}\left(t_{2}\right)\to \min\left(P_{z1}\left(t_{1}\right),P_{z2}\left(t_{2}\right)\right)$$

As such, if the *equivalent trajectory condition* is applied to the relevant segments of the trajectories given by Equations (7)–(10), then a trajectory called equivalent trajectory is created, as schematically illustrated in Figure 4. The concept of equivalent trajectory can be extended for multiple grains to determine the grinding effect on the workpiece surface.

### 4.3. Effect of Several Grains

**Figure 5:**
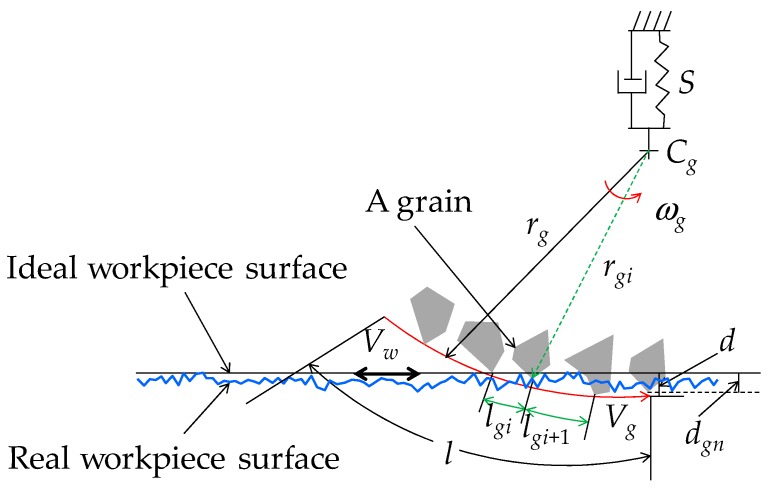
Microscopic interaction between abrasive grain and workpiece.

As mentioned in the previous subsection, the idea of the equivalent trajectory can be extended to see the effect of several grains. In this case, the random distribution, truncation, heights, and other real-life factors as described in Section 2 can be considered. In doing so, the schematic diagram shown in Figure 5 can be used to describe the microscopic interactions between the grinding wheel and workpiece surface.

As shown in Figure 5, let *l* and *m* be the length of a segment on the outermost circumference of the grinding wheel and the average grain population (i.e., the expected number of abrasive grains in *l*), respectively. The expected distance between two consecutive abrasive grains is given as follows:

(12) $$l_{g}=\frac{l}{m}$$

In reality, the actual number of grains, denoted as *n*, is most likely *n* ≠ *m*. In addition, the circumferential distance between two consecutive abrasive grains, denoted as *l_gi_* (*i* = 1, ..., *n*), exhibits a certain degree of stochasticity, i.e., it is most likely that *l_gi_* ≠ *l_g_*, ∀*i* ∈ {1, …, *n*}. The unimodal distributions (e.g., uniform/normal/triangular distributions) can be employed to generate *l_gi_* and then identify the value of *n* in a stochastic manner. As such, let *N*(*l_g_*,*l_g_*/*c*) be a normally distributed variable with mean *l_g_* and standard deviation *l_g_*/*c* (where *c* > 0 is the confidence interval). Therefore, the distance between two consecutive abrasive grains is given as follows:

(13) $$l_{gi}=\underset{}{\max}\left(0,N\left(l_{g},\frac{l_{g}}{c}\right)\right)$$

Moreover, the cumulative length denoted as *L_gi_*, up to the *i*-th grain in *l* can be calculated as follows:

(14) $$L_{gi}=\overset{i}{\underset{i=1}{\sum }}l_{gi}$$

Since the cumulative length *L_gi_* cannot be longer than *l*, the actual number of grains *n* is determined by the following logical expression:

(15) $$\left(L_{gi}\leq l\right)\wedge \left(L_{gi+1}>l\right)\to n=i$$

The logical expression in Equation (15) simply means that the index *i* can be increased as long as the condition *L_gi_* ≤ *l* is true; the final value of *i* for which this condition is still true is the actual number of grains, *n*.

Regarding the variability in the depth of cut of an abrasive grain as schematically illustrated in Figure 5, the following considerations can be made. Let *d* be the expected depth of cut. An abrasive grain may not attain this depth of cut, as it might have been truncated, worn, or even lost during the grinding operation. Therefore, the distance between the outermost cutting point of an abrasive grain and the center of the grinding wheel (*C_g_*), denoted as *r_gi_*, is most likely to be less than *r_g_*, i.e., *r_gi_* ≤ *r_g_*. Therefore, the actual depth of cut of an abrasive grain, denoted as *d_gi_*, is randomly distributed in the following interval:

(16) $$d_{gi}=\left[a,d\right]\;a\geq 0$$

Alternatively, the actual depth of cut can be modeled by using a normally distributed variable. In this case, if *N*(*µ_i_*,*σ_i_*) denotes a normally distributed variable with mean *µ_i_* and standard deviation *σ_i_*, then the actual depth of cut is given as follows:

(17) $$d_{gi}=\underset{}{\max}\left(0,N\left(\mu_{i},\sigma_{i}\right)\right)$$

Therefore, the actual radius of the grinding wheel for a grain is given as follows:

(18) $$r_{gi}=r_{g}-\left(d-d_{gi}\right)$$

As a result, the attack angle and the velocity of a grain are as follows:

(19) $$\theta_{gi}=2\left(\sqrt{\frac{2d_{gi}}{r_{gi}}}\right)$$

(20) $$V_{gi}=\omega_{g}r_{gi}$$

Similar to Equation (2), the contact period of a grain is given as follows:

(21) $$T_{gi}=2\frac{\sqrt{2d_{gi}r_{gi}}}{V_{gi}}$$

Similar to Equations (7) and (8), the coordinates of the trajectory of a grain are given as follows:

(22) $$P_{xi}\left(t\right)=x_{c}-r_{gi}\sin\left(\theta_{gi}-\omega_{g}t\right)+V_{w}\left(T_{gi}-t\right)+\overset{i-1}{\underset{i=1}{\sum }}l_{gi}V_{ri}$$

(23) $$P_{zi}\left(t\right)=z_{c}-r_{gi}\cos\left(\theta_{gi}-\omega_{g}t\right)$$

Since *V_ri_* = *V_w_*/*V_gi_* = *V_w_*/*ω_r_r_gi_* = (*V_w_*/*V_g_*) × (*r_g_*/*r_gi_*) = *V_r_*(*r_g_*/*r_gi_*), the *x*-coordinate of a grain can be written as follows:

(24) $$P_{xi}\left(t\right)=x_{c}-r_{gi}\sin\left(\theta_{gi}-\omega_{g}t\right)+V_{w}\left(T_{gi}-t\right)+V_{r}r_{g}\overset{i-1}{\underset{i=1}{\sum }}\frac{l_{gi}}{r_{gi}}$$

Let *P_j_* and *P_k_*, ∀*j* ∈ {1, …, *n*} and ∀*k* ∈ {1, …, *n*} − {*j*}, be two of the trajectories defined in Equations (22) and (23). If the equivalent trajectory condition as shown in Equation (11) is applied to the relevant segments of *P_j_* and *P_k_*, then the following relationship holds:

(25) $$P_{xj}\left(t_{j}\right)=P_{xk}\left(t_{k}\right)\to min\left(P_{zj}\left(t_{j}\right),P_{zk}\left(t_{k}\right)\right)$$

**Figure 6:**
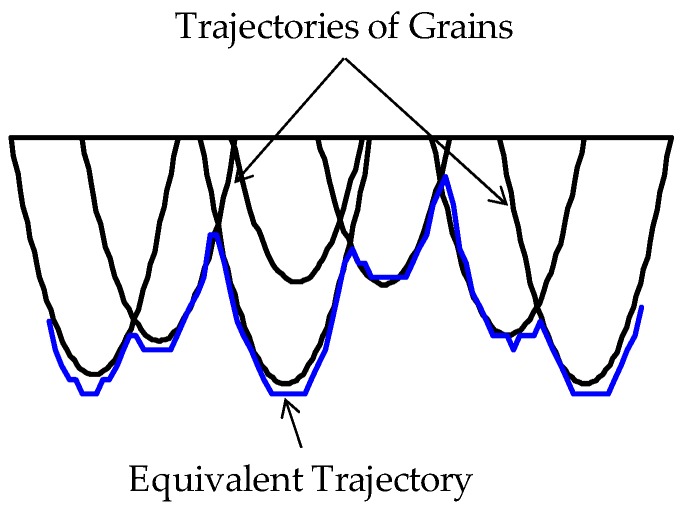
Ground surface generated by the resultant of multiple trajectories.

**Figure 7:**
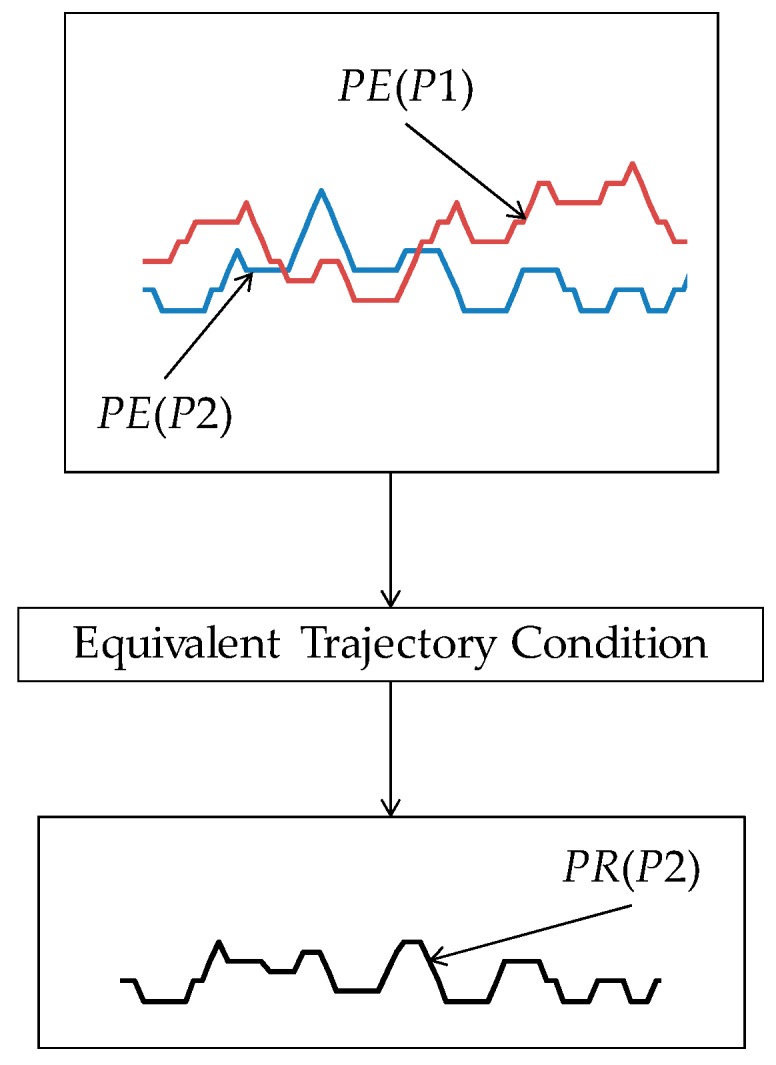
Creating resultant trajectory for two passes.

Note that in Equation (25), *t_j_* ∈ [0, *T_gj_*] and *t_k_* ∈ [0, *T_gk_*]. Figure 6 schematically illustrates the trajectories of a set of grains (seven grains as shown in Figure 6) and their combined effect, i.e., the equivalent trajectory. The equivalent trajectory is created by applying the equivalent trajectory condition (Equation (25)) to the trajectories defined in Equations (22)–(24). The equivalent trajectory is responsible for the surface roughness on the ground surface. If the grinding wheel passes the same area on the workpiece surface several times, the equivalent trajectory of a pass interacts with that of the previous pass. Let *PE*(*P*1) = (*PE_x_*(*P*1), *PE_z_*(*P*1)) be the equivalent trajectory of the first pass and *PE*(*P*2) = (*PE_z_*(*P*2), *PE_z_*(*P*2)) be the equivalent trajectory of the second pass. Here, *PE*(*P*1) and *PE*(*P*2) are determined using the formulation defined in Equations (22)–(25). The resultant trajectory *PR*(*P*2) generated after the second pass is created by applying the equivalent trajectory condition, as schematically illustrated in Figure 7.

The process can be defined by the logical expression as follows:

(26) $$PE_{x}\left(P1\right)=PE_{x}\left(P2\right)\to min\left(PE_{z}\left(P1\right),PE_{z}\left(P2\right)\right)\overset{yields}{\to }\;PR\left(P2\right)$$

Note that by default, *PE*(*P*1) = *PR*(*P*1). The logical process defined in Equation (26) can continue for the third pass, fourth pass, and so on.

**Figure 8:**
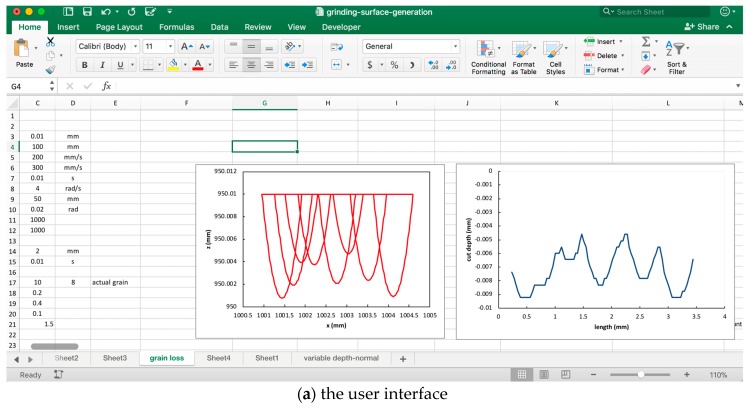

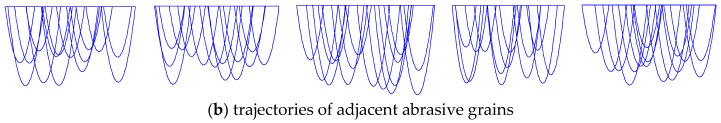
Grinding mechanism system.

**Figure 9:**
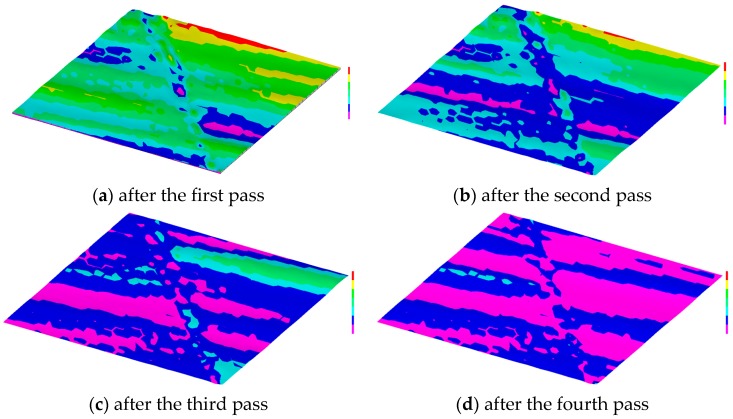
Theoretical surface topography of workpiece due to grinding.

A computer-based system has been developed that runs based on the mathematical procedures defined in Equations (1)–(25) and creates the equivalent trajectory according to Equation (26) (Figure 6). The user interface of this system is shown in Figure 8. As seen from Figure 8, the system displays the trajectories of some grains and the equivalent trajectory for different passes. The system is capable of calculating the stochastic parameters (e.g., the radius of a grain, distance between two consecutive grains, and the number of grains) while determining the trajectories using Monte Carlo Simulation [34]. One can easily create the trajectories of the adjacent abrasive grains using the system, as shown in Figure 8b. The *x*- and *z*-coordinates of the equivalent trajectories calculated by the system can be used to create a theoretical grinding surface, i.e., the resultant trajectory after each pass denoted as *PR*(*P*1), *PR*(*P*2), … While setting the parameters (e.g., average number of grains) one can use the nominal data of grinding wheel parameters. The system shown in Figure 8 is used to study the effect of multiple passes on the ground surface topography. The equivalent trajectories of successive and adjacent abrasive grains can be used to simulate the surface topography of a ground surface. One of the sets of results is shown in Figure 9. The yellow, red, and light green areas are the shallow areas whereas the blue and purple areas are the deep areas. As seen from Figure 9, the deep areas gradually increase with the increase in the number of passes. This means that the more the number of passes, the more the materials removed from the workpiece surface. A somewhat similar trend is seen in the real-life case as shown in Figure 2, however.

Now, while creating the topography shown in Figure 9, the center of the grinding wheel *C_g_* = (*x_g_*,*z_g_*) is kept constant, i.e., the machine stiffness (denoted as *S* as shown in Figure 5) or vibration is not considered. In real-life grinding, vibration is an integrated part of grinding. Moreover, the alignment and form errors of the grinding wheel and workpiece surface are not considered while creating the while creating the topography shown in Figure 9. In real-life grinding, the alignment and form errors of the grinding wheel and workpiece surface are the integrated part of grinding. Therefore, the real-life workpiece surface topography may not perfectly resemble the ones shown in Figure 9. For this reason, the differences between theoretical and experiment surface topographies should be compared to see whether or not the overall trend (i.e., the amount of material removed increases with the increase in the number of passes) is violated. This issue is described in details in the next section using the experimental results.

## 5. Experimental Investigation

This section describes the experiments performed to obtain the pass-by-pass workpiece surface topography due to grinding.

**Figure 10:**
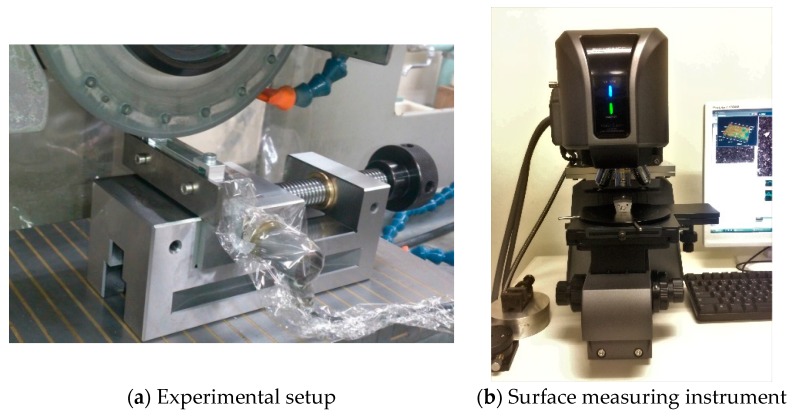
Experimental setup and surface measuring instrument.

**Figure 11:**
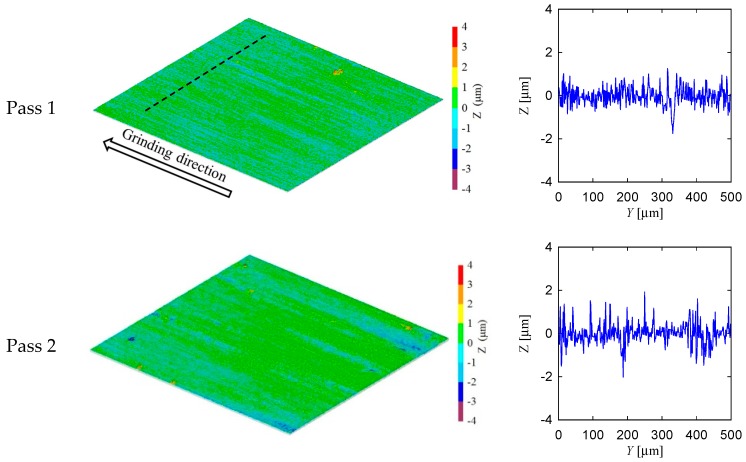

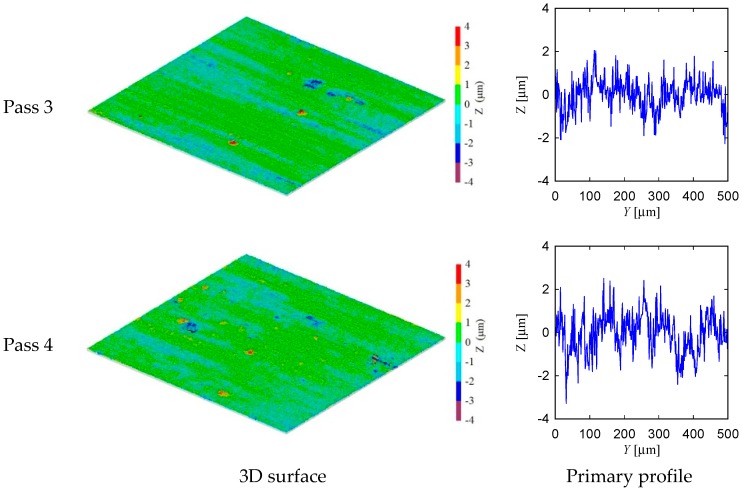
Surface topography of ground surface for feed rate *V_w_* = 40 mm/s.

**Figure 12:**
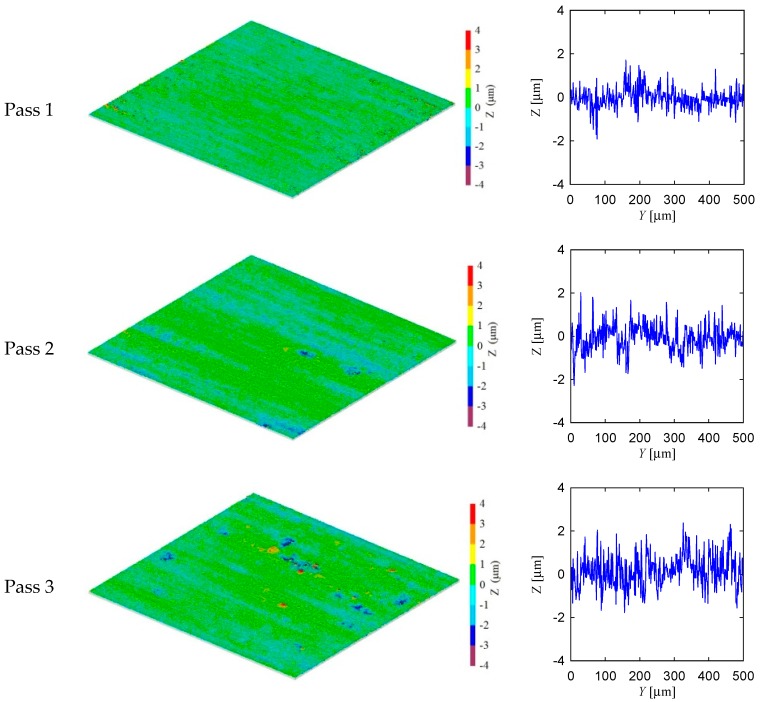

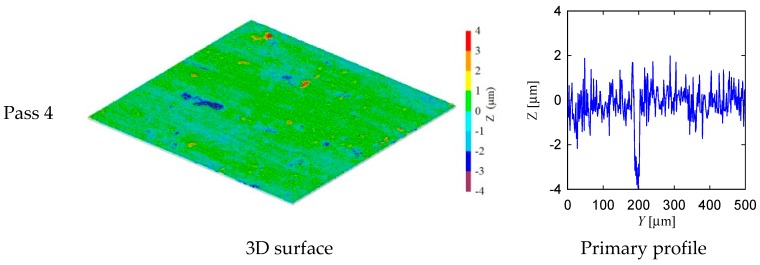
Surface topography of ground surface for feed rate *V_w_* = 100 mm/s.

Figure 10 shows the experimental setup and surface measuring instrument used in this section. Referring to Figure 10, the grinding machine and surface measuring instrument are described in Table 1. The specifications of the grinding wheel, coolant, and workpiece are also shown in Table 1. The grinding conditions are also shown in Table 1. Figure 11 and Figure 12 show the surface topography of the ground surfaces measured by a laser microscope (Table 1 and Figure 10b). For each pass, the primary profile of the ground surface is also shown in Figure 11 and Figure 12.

**Figure 13:**
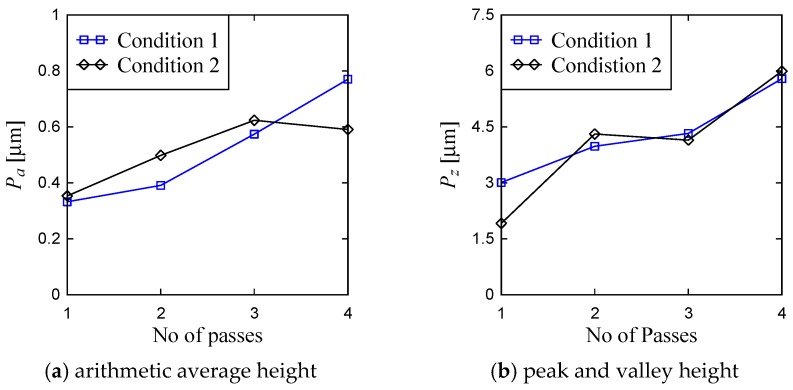
Roughness of the primary profiles.

From the visual inspection of the surface topography and primary profile, it is clear that the complexity of the ground surface increases with the increase in the number of passes. For the sake of quantification, the primary profile data is processed in both conventional and non-conventional ways, as described in [35,36]. As a part of conventional way, the surface roughness parameters denoted as *P_a_* (arithmetic average height) and *P_z_* (peak and valley height) are calculated using the surface height data points as shown in Figure 11 and Figure 12. (Note that when the arithmetic average height and peak and valley height are determined from the roughness profile they are denoted as *R_a_* and *R_z_*, respectively.) The results are shown in Figure 13. In Figure 13, the Condition 1 means the feed rate is equal to 40 mm/s and Condition 2 means the feed rate is equal to 100 mm/s. The other cutting conditions are the same for both Conditions 1 and 2, as shown in Table 1. For both conditions, the trend remains the same, i.e., an increase in the number of passes increases the *P_a_* and *P_z_*. However, there is an exception. For Condition 2, *P_a_* obtained after the third pass is greater than that of after the fourth pass. In addition, *P_z_* remains almost the same for the second and third passes for Condition 2.

**Figure 14:**
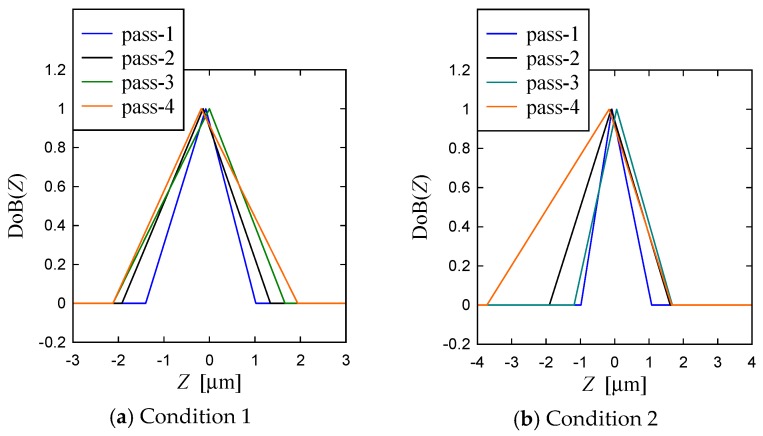
Possibility distributions of the surface heights of ground surface due to multiple passes.

As a part of non-conventional quantification, the possibility distributions (probability distribution neutral representation of uncertainty) of the profile heights are also constructed, as shown in Figure 14, using the possibility distribution determination process described in Ullah and Shamsuzzaman [34] and Chowdhury et al. [37]. In Figure 14, the phrase “DoB” means the Degree of Belief. As seen from Figure 14, the primary profile heights about zero have very high possibility (DoB ≅ 1) whereas the possibility gradually decreases if the heights move away from the zero. For both conditions the possibility (or DoB) of variability in the surface heights increases with the increase in the number of passes. This means that the height distributions become more random due to the increase in the number of passes.

**Figure 15:**
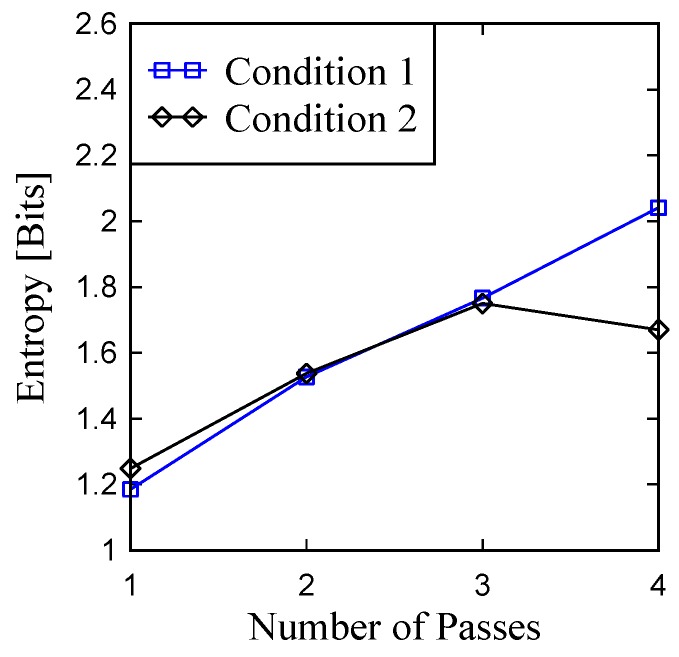
Entropy of the ground surfaces due to multiple passes.

To be more specific, the entropy (or average information content) of the primary profile is determined using the procedure described in Ullah et al. [35]. The results are shown in Figure 15. For Condition 1, the entropy gradually increases from about 1.2 Bits to 2.0 Bits, whereas this trend is true from the other conditions except for the last pass where the entropy decreases compared to that of the previous pass. Since the more the entropy, the more the complexity, the ground surface tends to become more and more complex due to the addition of passes.

## 6. Concluding Remarks

This study sheds some lights on the theoretical and experimental understanding of how a grinding wheel removes materials and leaves roughness on the workpiece surface due to the multiple passes. The following conclusions can be made based on the outcomes of this study.

The literature review on grinding mechanism shows that a microscopic interaction between the abrasive grains (attached on the outermost circumference of a grinding wheel) and workpiece surface takes place in the presence of the following conditions: loss/truncation/wear of abrasive grains, uneven size, height, and distribution of abrasive grains, roughness of the already-ground workpiece surface, thermal/elastic/plastic deformations, machine/grinding wheel stiffness, and alignment and form errors of the workpiece and grinding wheel. This microscopic interaction is the cause of the complex surface topography.

A grinding mechanism model is developed to predict the workpiece surface topography accommodating the following conditions: loss/truncation/wear of abrasive grains, uneven size, height, and distribution of abrasive grains, and roughness of the already-ground workpiece surface. A computing tool is also developed implementing the model. The workpiece surface topography is determined by using the computing tool for multipass grinding. It is found that the more the number of passes, the more the materials removed from the workpiece surface. It is also found that the more the number of passes, the more the complexity of the ground surface.

The theoretical grinding mechanism model does not incorporate some of the realistic factors (i.e., the thermal/elastic/plastic deformations, machine/grinding wheel stiffness, and alignment and form errors of the workpiece and grinding wheel). Thus, multipass grinding experiments are conducted to see whether or not the conclusion drawn from the theoretical model hold in the real-life settings. It is found that the topography of the ground surface due to multipass grinding resembles the theoretical topography. The conventional parameters (the arithmetic average height and the peak and valley height) as well as such unconventional parameters (possibility distribution and entropy) of primary profile of the ground surface are used to quantify the complexity of the surface topography for multipass grinding. 

Therefore, compared to other real-life factors, (1) the loss/truncation/wear of abrasive grains; (2) the uneven size, height, and distribution of abrasive grains; and (3) the roughness of the already-ground workpiece surface are the main factors that govern the grinding mechanism (i.e., the microscopic interaction between the abrasive grains attached on the outermost circumference of a grinding wheel and workpiece surface).

Nevertheless, the gap between the analytical and experimental results might be used to develop a more comprehensive grinding mechanism model. This issue remains open for further research.

## Authors Contributions

AMM Sharif Ullah, Alessandra Caggiano, Akihiko Kubo, and M. A. K. Chowdhury conceived the idea. AMM Sharif Ullah, Alessandra Caggiano, and Akihiko Kubo worked on the introduction section. AMM Sharif Ullah, Alessandra Caggiano and M. A. K. Chowdhury worked on the literature review section. AMM Sharif Ullah and Akihiko Kubo worked on the preliminary experimentation section. AMM Sharif Ullah, Alessandra Caggiano, Akihiko Kubo worked on the experimental work section. AMM Sharif Ullah, Alessandra Caggiano, Akihiko Kubo, and M. A. K. Chowdhury worked on the experimental investigation. AMM Sharif Ullah and Alessandra Caggiano worked on the concluding remarks section. AMM Sharif Ullah and Alessandra Caggiano wrote the paper.

## Figures and Tables

**Table 1:** Experimental Conditions.

Grinding Machine	Surface Grinding Machine, PSG-52DX made by Okamoto Machine Tool Works Ltd. (Gunma, Japan)
Grinding wheel	CBN170M100B Diameter *D* = 180 mm, Width *B* = 10 mm
Workpiece	Ordinary Glass
Grinding conditions	Rotational speed of grinding wheel, *N* = 300 rpm Workpiece speed (feed rate) *V_w_* = 40, 100 mm/s Depth of cut, *d* = 6 μm Down cut
Coolant	Chemical Solution WS90 made by Yushiro Chemical Industry Co. Ltd. (Tokyo, Japan)
Measuring Instrument	Laser Scanning Microscope VK-9700 made by Keyence Corporation (Osaka, Japan)
